# Supplementary material for: Mast Cells Limit Ear Swelling Independently of the Chymase Mouse Mast Cell Protease 4 in an MC903-Induced Atopic Dermatitis-Like Mouse Model
Source: Int J Mol Sci. 2020 Aug 31;21(17):6311. doi: 10.3390/ijms21176311 (PMC7503626; doi:10.3390/ijms21176311)
Supplement: Supplementary file 1 [file ijms-21-06311-s001.pdf]

Supplementary data and figures.

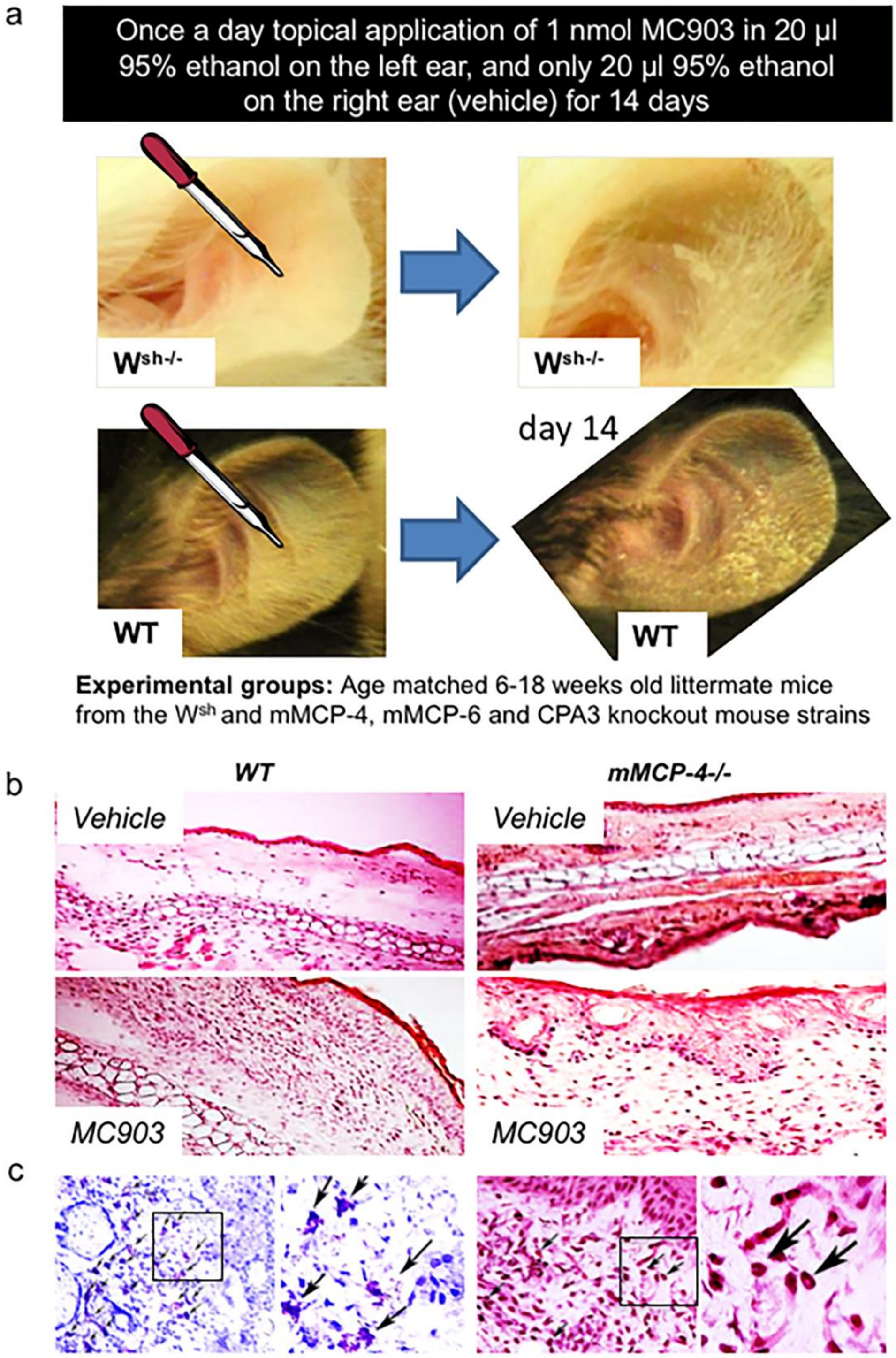

Supplementary Figure 1. MC903 treatment induce clinical symptoms and granulocyte and mast cell infiltration in the ear tissue. a) The experimental model with 1nmol MC903 in 20µl of ethanol. **b)** H&E staining of ear tissue sections in WT (left panel) and mMCP4$^{-/-}$ (right panel) with vehicle or MC903 application for 14 days shows similar inflammation in both genotypes. **c)** Left panels with inset (inset framed in the micrograph) shows TB-stained mast cell infiltration. Right panel (H&E) with inset (inset framed in the micrograph) shows granulocyte infiltration. Representative photographs are shown.

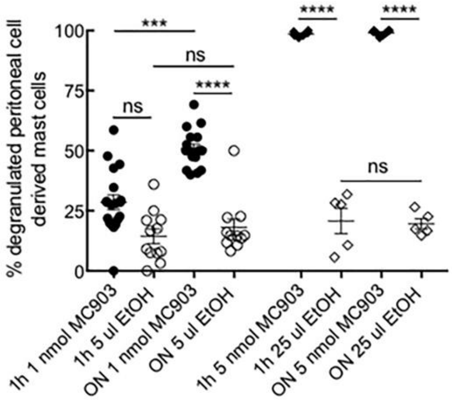

Supplementary Figure 2. MC903 induce activation and degranulation of peritoneal cell derived mast cells (PCMCs). Both 1nmol and 5 nmol MC903 induced degranulation of peritoneal derived mast cells when stimulated for 1hr or overnight (ON) in in vitro cell cultures. Stimulation with ethanol alone used as a control (vehicle) had little effect. PCMCs were derived from 4 individual WT mice and PCMCs were challenged in quadruplicates with the 1 nmol MC903 concentration and in triplicates for the vehicle (5 µl EtOH), or in duplicates for the 5 nmol MC903 concentration. *** P <0.00, **** P <0.0001, ns, not significant.

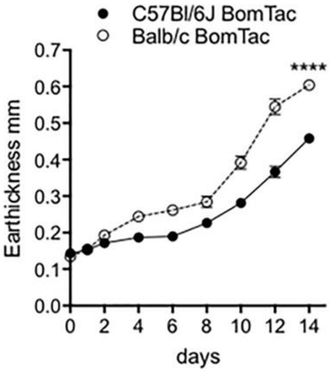

Supplementary Figure 3. Genetic background of the mice impact on earthickness in MC903-induced atopic dermatitis. 12-18 weeks old Balb/c Taconic (BomTac) mice (n=6) shows a significantly increased earthickness compared to C57BL/6J Taconic (n=6) mice following topical application of MC903 for 14 days. A statistically significant increase in earthickness in the Balb/c mice was observed from day 4 and onward. **** P <0.0001.
